# Inverting the Concentrations of Chlorophyll-a and Chemical Oxygen Demand in Urban River Networks Using Normalized Hyperspectral Data

**DOI:** 10.3390/s25227004

**Published:** 2025-11-16

**Authors:** Rongda Guan, Yingzhuo Hou, Maham Arif, Qianguo Xing

**Affiliations:** 1 Shandong Key Laboratory of Coastal Zone Environmental Processes and Ecological Security, Yantai Institute of Coastal Zone Research, Chinese Academy of Sciences, Yantai 264003, China; rdguan@yic.ac.cn (R.G.); mahamarif@yic.ac.cn (M.A.); 2 University of Chinese Academy of Sciences, Beijing 100049, China

**Keywords:** hyperspectral sensor, remote sensing, machine learning, chlorophyll-a, chemical oxygen demand, Zhongshan river networks

## Abstract

Chlorophyll-a (Chl-a) and chemical oxygen demand (COD) are key indicators for water quality evaluation. In previous research on the inversion of Chl-a and COD concentrations using hyperspectral data, disparities in hyperspectral data types have constrained the universality of the inversion models. To solve this problem, in this study, synchronous in situ hyperspectral data and water samples were collected from 308 stations within the river networks of Zhongshan City. Four inversion models, support vector regression (SVR), random forest (RF), backpropagation neural network (BPNN), and one-dimensional convolutional neural network (1D-CNN), were established using the original reflectance (R), remote sensing reflectance (Rrs), and their normalized forms as inputs. To evaluate the robustness of the models, their performance was assessed via cross-reflectance type validation. For example, a model was trained using R data and then tested with Rrs data. The results show that using the normalized hyperspectral data for modeling not only improves the accuracy of the inversion results of Chl-a and COD concentrations, but also effectively unifies different types of hyperspectral data, thereby improving the versatility of the inversion model. This study provides a reference for constructing a general water quality inversion model based on hyperspectral data.

## 1. Introduction

The normal functioning of a city is inseparable from water resources, and the quality of water is closely intertwined with the daily lives of humans [1]. With the development of cities, the pollution problem of urban rivers has become increasingly serious, which makes water quality monitoring more and more important [2,3,4,5]. Traditional water quality monitoring methods rely on field sampling and laboratory analysis, which are costly, time consuming, and may even cause secondary pollution, making it difficult to achieve long-term monitoring [6,7]. In recent years, remote sensing technology has begun to be applied to water quality monitoring. This non-contact monitoring method evaluates water quality by analyzing the spectrum of the obtained water body [8,9,10,11]. Compared with traditional water quality monitoring methods, the remote sensing-based monitoring method has more advantages in terms of spatial scale and is less costly [12,13].

Chlorophyll-a (Chl-a) and chemical oxygen demand (COD) are crucial indicators for water quality evaluation and significant water quality parameters [14,15]. Chl-a is a green pigment widely present in the cells of algae, plankton, etc., and is an important part of photosynthesis [16]. The content of Chl-a can reflects the richness of the biomass in water [17,18]. COD refers to the substances in water that can be chemically oxidized under certain conditions. Its concentration can reflect the content of reducing substances in water and is regarded as an important water quality parameter representing the degree of organic pollution [19,20,21]. Since different components in water selectively absorb or reflect electromagnetic waves from diverse light sources, remote sensing devices can quantitatively analyze the concentrations of various components in water by detecting the intensity of electromagnetic waves reflected from the water surface [22,23]. Currently, numerous studies have utilized various remote sensing devices to monitor the concentrations of various components in water, such as Chl-a and COD [24].

As common remote sensing devices, hyperspectral sensors capture the digital number values (DN) of target objects. To ensure data reliability and accurate estimation of water quality parameters, DN values are typically not used to construct inversion models [25,26,27]. Remote sensing reflectance (Rrs) is defined as the ratio of the water-leaving radiance to the downwelling irradiance [28], and reflectance (R) is defined as the ratio of the upwelling irradiance to the downwelling irradiance [29,30]. However, in the actual above-water measurement method, since the hyperspectral sensor cannot directly obtain the downwelling irradiance of the water surface, R and Rrs are usually obtained indirectly by measuring the signal of the standard plate [31,32]. For example, Jia et al. used the standard plate to calculate Rrs of the water body in Hangzhou Bay [33], and Ma and Dai used the standard plate to calculate R of the water body in Meiliang Bay, Taihu Lake [34]. At present, many algorithms based on R and Rrs have been developed to estimate the concentrations of Chl-a and COD. Machine learning algorithms offer superior fitting ability and feature learning, leading to their increased use in inverting concentrations of water quality parameters like Chl-a and COD [35,36,37,38,39]. For example, Zhu et al. used Rrs to construct support vector regression model to estimate the Chl-a concentration [40], Sun et al. used Rrs to construct back propagation neural network to estimate the concentrations of Chl-a and COD [41], and Cai et al. used R to construct a one-dimensional convolutional neural network to estimate the COD concentration [42].

In addition to directly using the original hyperspectral data to establish inversion models as in the above-mentioned studies, there are also studies that preprocess the original hyperspectral data before establishing inversion models. Hyperspectral normalization processing can effectively remove the influence of factors such as the environment and measurement angle on the hyperspectral sensor when measuring water bodies, and there have been studies using normalized hyperspectral data to establish water quality inversion models [43,44]. However, the models constructed in these studies are all based on the single type of hyperspectral data obtained by themselves [40,41,42,43,44]. These studies did not explore whether the normalization processing can improve the performance of the inversion model, nor did they explore whether this processing can integrate different types of hyperspectral data. Due to the differences in the measurement and calculation methods of Rrs and R, the model established with a single type of hyperspectral data set may not be applicable across all scenarios. Consequently, to investigate if normalization can create a universal water quality inversion model applicable to both Rrs and Rr, this study separately utilized R and Rrs to construct machine learning models for inverting the concentrations of Chl-a and COD. Subsequently, the performance of these models was tested using the other type of hyperspectral data. This study aims to offer insights for establishing a general water quality inversion model.

## 2. Data and Methods

### 2.1. Study Area

**Figure 1:**
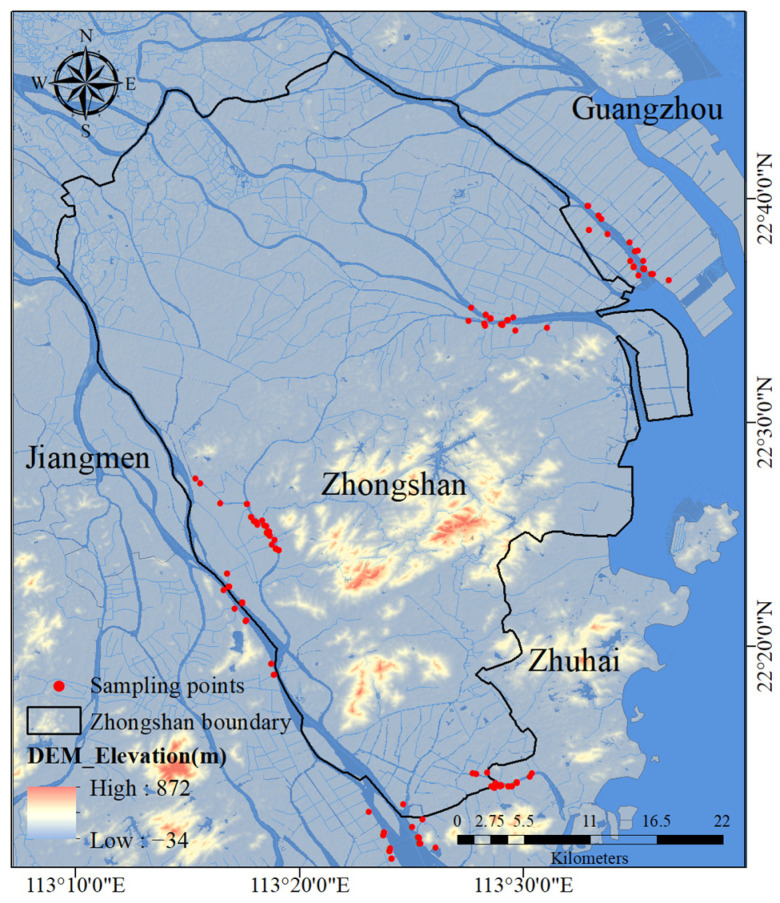
Study area and sampling point distribution.

Zhongshan City, located in the central-southern part of Guangdong Province, China, is one of the key constituents of the Guangdong–Hong Kong–Macao Greater Bay Area. With over 1000 rivers and drainage ditches of various scales, Zhongshan ranks among the regions with relatively high river networks density in China [45,46]. As illustrated in Figure 1, the sampling sites of this study are primarily concentrated in Zhongshan river networks and its adjacent major water areas.

### 2.2. Water Sample Collection and Hyperspectral Data Preprocessing

Water samples and supporting hyperspectral data were collected from 9 July 2019, to 30 June 2020, and a total of 308 sets of data were collected. The distribution of sampling points is shown in Figure 1. The Chl-a concentration was measured using the spectrophotometric method (HJ897—2017 [47]), and the COD concentration was measured using the dichromate method [48]. The measurement results of water samples are shown in Table 1. Rrs and R were measured using the above-water measurement method, and the measurement equipment was a USB4000 spectrometer. This equipment was developed by Ocean Optics in Dunedin, Florida, USA, and its spectral measurement range is 345.3–1046.12 nm, and the sampling interval is approximately 0.2 nm. To ensure the accuracy of the measurement data, at each sampling point, three rapid and continuous measurements were made of the water surface-reflected radiation, the reflected radiation of the reflectance standard plate, and the sky radiation. The type of calibration plate is diffuse reflection calibration plate. To mitigate the impact of environmental variations on the measurements, this study opted to carry out measurements on sunny and cloudless days. During each measurement, the equipment probe was positioned approximately 0.5 m away from both the water body and the standard plate, ensuring that neither the water body nor the standard plate was obscured by shadows. The calculation formulas for R and Rrs [31,32] are shown as follows:

(1) $$Rrs=\rho_{p}\times \frac{S_{sw}-r\times S_{sky}}{\pi \times S_{p}}$$

(2) $$R=\rho_{p}\times \frac{S_{sw}}{S_{p}}$$

where $\rho_{p}$ is the reflectance of the standard plate with a value of 0.25, r is the Fresnel reflectance of the skylight at the water surface with a value of 0.028 [31], and $S_{sw}$, $S_{sky}$, and $S_{p}$ are the measurement DN values when the spectrometer faces the water body, the sky, and the standard plate. To minimize errors in the measurement process, we calculate the average values of the Rrs and R obtained from the three measurements separately. These average values are then utilized as the actual values for this set of Rrs and R. To simplify subsequent modeling and remove data redundancy caused by redundant bands, in this study, the interp1 function in MATLAB R2023a was used to linearly interpolate the calculated Rrs and R spectra and resample them to a resolution of 1 nm.

**Figure 2:**
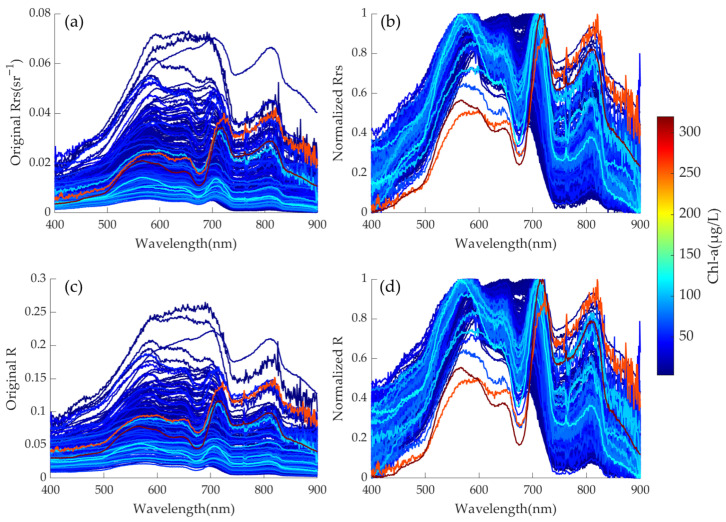
(**a**,**b**) in situ Rrs (by Formula (1)) and normalized Rrs (by Formula (3)), and (**c**,**d**) in situ R (by Formula (2)) and normalized R (by Formula (4)). The color bar on the right indicates the Chl-a concentration corresponding to the color of each spectrum.

In this study, the method of using minimum–maximum normalization to process the original hyperspectral data was used to preprocess Rrs and R. The calculation formula is as follows:

(3) $${Rrs}_{\lambda i}^{N}=\frac{{Rrs}_{\lambda i}-\min\left(Rrs\right)}{\max\left(Rrs\right)-\min\left(Rrs\right)}$$

(4) $$R_{\lambda i}^{N}=\frac{R_{\lambda i}-\min\left(R\right)}{\max\left(R\right)-\min\left(R\right)}$$

where λi is the wavelength between 400 and 900 nm, ${Rrs}_{\lambda i}$ is Rrs at λi, $R_{\lambda i}$ is R at λi, min(Rrs) and max(Rrs) are the minimum and maximum values of Rrs in the range of 400–900 nm, min(R) and max(R) are the minimum and maximum values of R in the range of 400–900 nm, ${Rrs}_{\lambda i}^{N}$ is the normalized Rrs at λi, and $R_{\lambda i}^{N}$ is the normalized R at λi. Finally, each sample in the sorted dataset contains 6 parameters: the concentrations of Chl-a and COD, along with their corresponding R, Rrs, normalized R and normalized Rrs. The spectrograms of Rrs and R and after normalization are shown in Figure 2.

### 2.3. Retrieval Models for Estimating Chl-a and COD Concentration

In this study, four common machine learning algorithms, namely support vector regression (SVR), random forest (RF), back propagation neural network (BPNN), and one-dimensional convolutional neural network (1D-CNN), were used to construct inversion models for Chl-a and COD concentrations. The above algorithms were implemented through the machine learning-related library functions provided by MATLAB R2023a.

SVR is an extension of Support Vector Machine (SVM) in regression problems [49]. Its core idea is to find an optimal function so that as many samples as possible fall within the preset error range, while minimizing the complexity of the function to ensure generalization ability [50]. In this study, the fitrsvm function was used to implement the SVR algorithm. The kernel function type was specified as the Radial Basis Function (RBF) suitable for processing non-linear relationships, and the scale parameter of the kernel function was set to be automatically calculated.

RF integrates the functions of the bagging algorithm, the random subspace algorithm, and the classification and regression tree [51,52]. By combining the prediction results of multiple decision trees and performing dual randomization of samples and features, the performance and robustness of the model are improved [53]. In this study, the templateTree and fitrensemble functions were used to implement the RF algorithm. The ensemble method was specified as bagging, and the minimum leaf node size of the decision tree was set to 5. 22 features that were used for node splitting based on random feature selection in each node and 100 trees were constructed per ensemble. In this study, no maximum tree depth was specified. As a result, the tree would keep growing until the sample size of the child nodes formed from the leaf nodes became less than 5.

BPNN is a multi-layer artificial neural network trained based on the backpropagation algorithm, mainly including an input layer, a hidden layer, and an output layer [54,55]. The input layer is used to receive the original data, and the number of neurons is equal to the dimension of the input features. The hidden layer is located between the input layer and the output layer and is responsible for extracting the abstract features of the data, which can include one or more layers. The output layer then outputs the prediction results, and the number of neurons is equal to the dimension of the task objective. The training process of BPNN is divided into a forward propagation stage of calculating the output and error and a backpropagation stage of calculating the gradient and updating the parameters, which iterates cyclically until the error converges [56]. In this study, the feedforwardnet function was used to implement forward propagation, and the trainlm function was used to implement backpropagation. The network structure includes two hidden layers (with 10 and 5 neurons, respectively), and the training loop is 1000 epochs.

**Figure 3:**
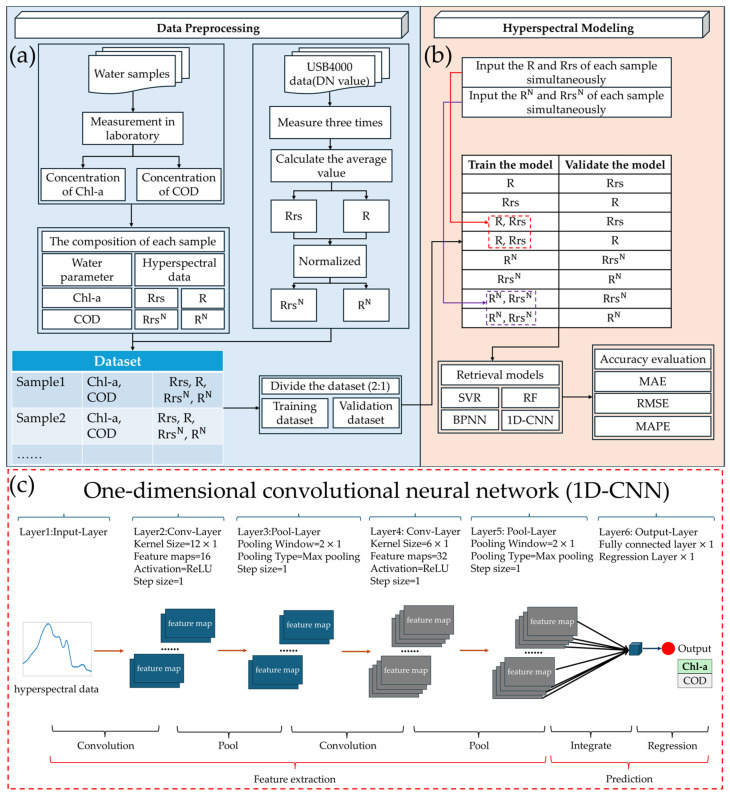
Technical flowchart of the framework for inverting Chl-a and COD concentrations based on machine learning. (**a**) Preprocess the measured data and partition the dataset; (**b**) use different types of hyperspectral data to train and validate the model. (**c**) The structural diagram of the 1D-CNN.

1D-CNN is a variant of the convolutional neural network (CNN) and is specifically designed to process one-dimensional sequence data [57,58]. It extracts local features in the sequence through a one-dimensional convolutional kernel, and while preserving the sequential order information of the sequence, realizes efficient feature learning. Similar to BPNN, 1D-CNN also includes an input layer and an output layer, and their functions are the same. The difference is that 1D-CNN usually also includes convolutional layers, pooling layers, and fully connected layers. The convolutional layer slides on the sequence through one-dimensional convolutional kernels to calculate and extract local features. The pooling layer is responsible for compressing the length of the feature sequence, reducing parameters and computational complexity, and at the same time, enhancing the robustness to local perturbations. The fully connected layer is responsible for mapping the feature sequence that has been compressed into a low-dimensional feature vector to the task objective [59]. In this study, the Deep Learning Toolbox provided by MATLAB was used to construct 1D-CNN. This neural network consists of an input layer, two convolutional layers, two pooling layers, a fully connected layer, and a regression output layer. In the first convolutional layer, the convolution kernel size is 12 × 1, while in the second convolutional layer, the convolution kernel size is 6 × 1. Each pooling layer has a pooling window of 2 × 1. The Rectified Linear Unit (ReLU) activation function is applied between convolutional and pooling layers. The network employs the Adaptive Moment Estimation (Adam) algorithm as the gradient descent method to update the model parameters. The training process is configured as cyclic training, running for 1000 epochs. The network structure is shown in Figure 3c.

The dataset was randomly divided into a training set and a validation set according to a ratio of 2:1. To enable the machine learning algorithm to make full use of all hyperspectral information, this study input the hyperspectral data corresponding to all bands in the range of 400–900 nm when training the model. To examine the impact of different types of hyperspectral data on the model performance, this study trained and validated the model using different types of hyperspectral data, respectively. For instance, one can train the model with R corresponding to the samples in the training set and subsequently verify the model’s performance using Rrs corresponding to the samples in the validation set. Alternatively, the model can be trained using both R and Rrs corresponding to the samples in the training set, and then its performance can be verified using R corresponding to the samples in the validation set. The modeling process is shown in Figure 3b.

The performance of each inversion model was evaluated by the Mean Absolute Error (MAE), Mean Absolute Percentage Error (MAPE), and Root Mean Square Error (RMSE), and the calculation formulas are as follows:

(5) $$MAE=\frac{\sum_{i=1}^{n}\left|y_{i}-y_{i}^{\prime }\right|}{n}$$

(6) $$MAPE=\frac{\sum_{i=1}^{n}\left|\frac{y_{i}-y_{i}^{\prime }}{y_{i}}\right|}{n}\times 100\%$$

(7) $$RMSE=\sqrt{\frac{\sum_{i=1}^{n}{\left(y_{i}-y_{i}^{\prime }\right)}^{2}}{n}}$$

where $y_{i}$ is the measured value, and $y_{i}^{\prime }$ is the value estimated by the model.

## 3. Results

### 3.1. Inversion Results of Chl-a Concentration

**Figure 4:**
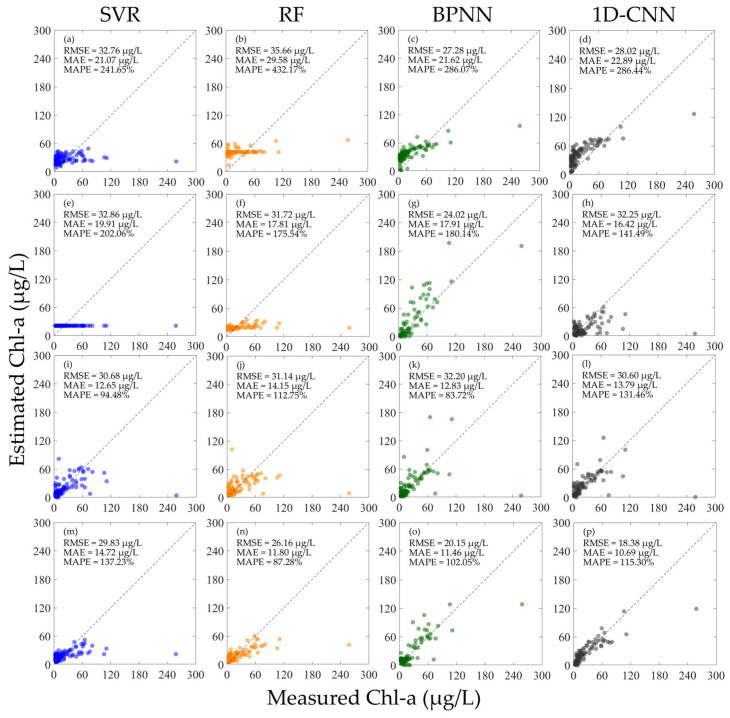
Results of constructing machine learning inversion models for Chl-a concentration using different types of hyperspectral data before normalization and validating the performance using different hyperspectral data. (**a**–**d**) Train the model using R and verify the model performance using Rrs; (**e**–**h**) train the model using Rrs and verify the model performance using R; (**i**–**l**) train the model using the mixed R and Rrs and verify the model performance using Rrs; and (**m**–**p**) train the model using the mixed R and Rrs and verify the model performance using R.

When the hyperspectral data is not normalized, each Chl-a concentration inversion model is trained and verified using different hyperspectral data, and the results are shown in Figure 4. The results show that whether using R or Rrs for training, the performance of the model is generally poor when using other hyperspectral data for verification. As shown in Figure 4a–h, the MAPE of all models is above 100%. When R and Rrs are mixed for training, the performance of each machine learning model is basically improved, especially for the SVR, RF, and BPNN models. Comparing all the results in Figure 4, the BPNN model achieved the best results when using the mixed R and Rrs for training. As shown in Figure 4k,o, when using Rrs for verification, the RMSE is 32.20 µg/L, the MAE is 12.83 µg/L, and the MAPE is 83.72%; when using R for verification, the RMSE is 20.15 µg/L, the MAE is 11.46 µg/L, and the MAPE is 102.05%. As shown in Figure 4i–p, although each model has better performance than using the separate R and Rrs for training in Figure 4a–h, the inversion performance of the model in the high concentration Chl-a region is still poor.

**Figure 5:**
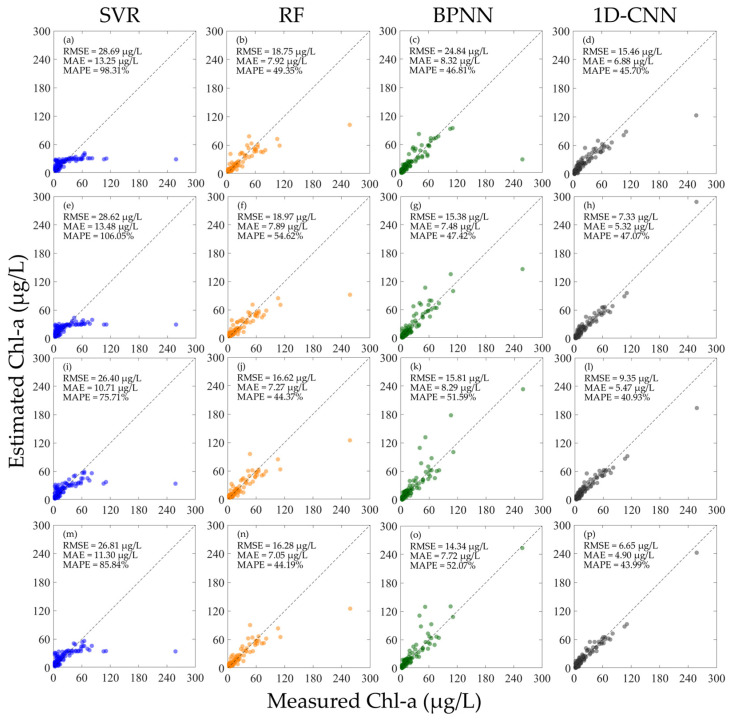
Results of constructing machine learning inversion models for Chl-a concentration using different types of normalized hyperspectral data and validating the performance using different hyperspectral data. (**a**–**d**) Train the model using $R^{N}$ and verify the model performance using ${Rrs}^{N}$; (**e**–**h**) train the model using ${Rrs}^{N}$ and verify the model performance using $R^{N}$; (**i**–**l**) train the model using the mixed $R^{N}$ and ${Rrs}^{N}$ and verify the model performance using ${Rrs}^{N}$; and (**m**–**p**) train the model using the mixed $R^{N}$ and ${Rrs}^{N}$ and verify the model performance using $R^{N}$.

As shown in Figure 5, after normalizing the hyperspectral data, the performance of each model has been significantly improved compared with Figure 4. When training the SVR, RF, and 1D-CNN models with mixed $R^{N}$ and ${Rrs}^{N}$, the models perform better than when training with separate $R^{N}$ and ${Rrs}^{N}$. However, for the BPNN, the converse is true. As shown in Figure 5c,g,k,o, although the overall performance of the BPNN when using the mixed $R^{N}$ and ${Rrs}^{N}$ for training is not as good as using the separate $R^{N}$ and ${Rrs}^{N}$, its accuracy is significantly improved when inverting the high concentration Chl-a. Comparing all the models in Figure 5, the 1D-CNN has the best performance under all training conditions. Among them, when using the mixed $R^{N}$ and ${Rrs}^{N}$ for training, the effect is the best. As shown in Figure 5l,p, when using the ${Rrs}^{N}$ for verification, the RMSE is 9.35 µg/L, the MAE is 5.47 µg/L, and the MAPE is 40.93%; when using the $R^{N}$ for verification, the RMSE is 6.65 µg/L, the MAE is 4.90 µg/L, and the MAPE is 43.99%.

### 3.2. Inversion Results of COD Concentration

**Figure 6:**
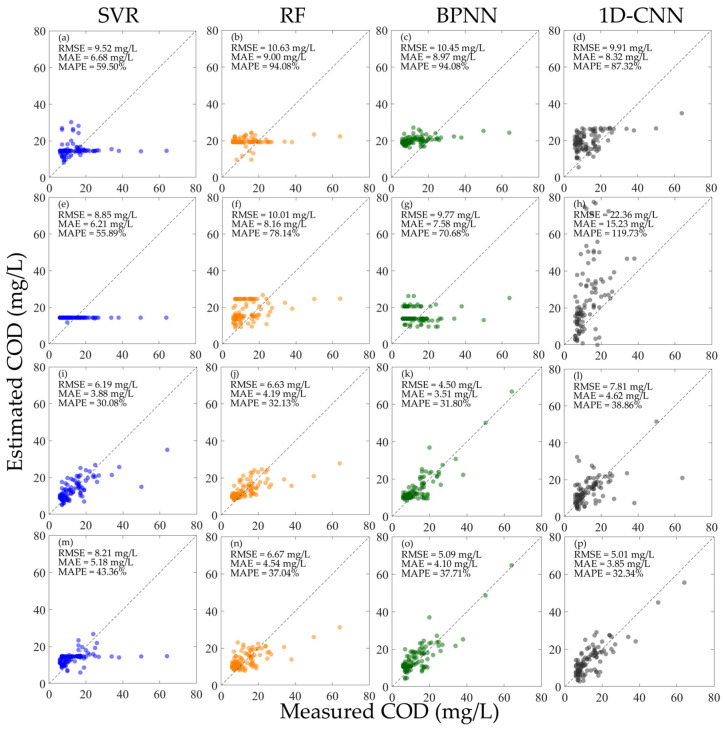
Results of constructing machine learning inversion models for COD concentration using different types of hyperspectral data before normalization and validating the performance using different hyperspectral data. (**a**–**d**) Train the model using R and verify the model performance using Rrs; (**e**–**h**) train the model using Rrs and verify the model performance using R; (**i**–**l**) train the model using the mixed R and Rrs and verify the model performance using Rrs; and (**m**–**p**) train the model using the mixed R and Rrs and verify the model performance using R.

As shown in Figure 6, when the hyperspectral data is not normalized, the performance of each model when using R or Rrs alone for training is lower than that when using the mixed R and Rrs for training. Among them, the SVR and RF have relatively low inversion accuracy for high concentration COD, while the BPNN and 1D-CNN have better effects. Overall, among all the models, the BPNN achieved the best results when using the mixed R and Rrs for training. As shown in Figure 6k,o, when using Rrs for verification, the RMSE is 4.50 mg/L, the MAE is 3.51 mg/L, and the MAPE is 31.80%; when using R for verification, the RMSE is 5.09 mg/L, the MAE is 4.10 mg/L, and the MAPE is 37.71%. Different from the BPNN, which also uses the mixed R and Rrs for training, the 1D-CNN has opposite results when using Rrs and R to verify the model performance, respectively, and the performance of using Rrs to verify the model is better than that using R for verification. As shown in Figure 6l,p, when using Rrs for verification, the RMSE is 7.81 mg/L, the MAE is 4.62 mg/L, and the MAPE is 38.86%; when using R for verification, the RMSE is 5.01 mg/L, the MAE is 3.85 mg/L, and the MAPE is 32.34%.

**Figure 7:**
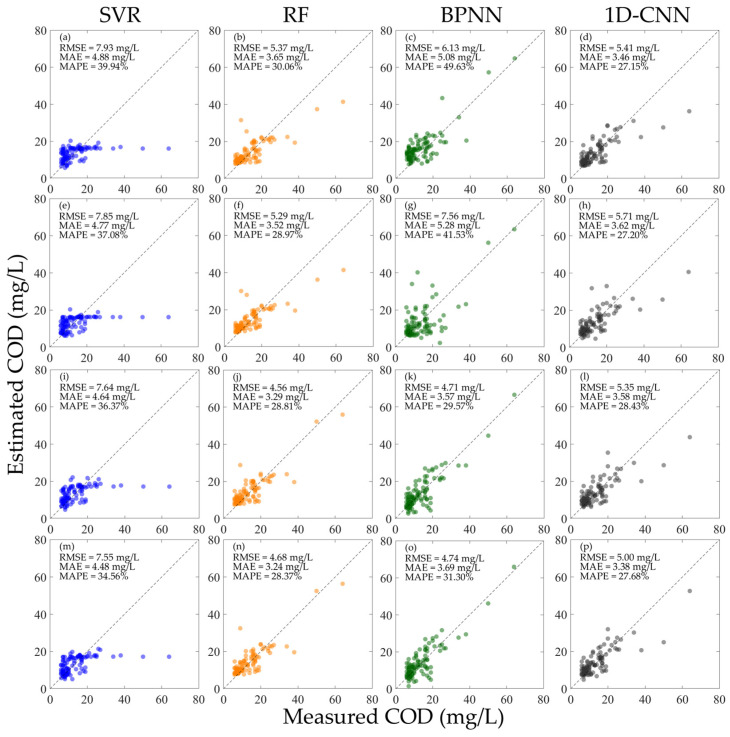
Results of constructing machine learning inversion models for COD concentration using different types of normalized hyperspectral data and validating the performance using different hyperspectral data. (**a**–**d**) Train the model using $R^{N}$ and verify the model performance using ${Rrs}^{N}$; (**e**–**h**) train the model using ${Rrs}^{N}$ and verify the model performance using $R^{N}$; (**i**–**l**) train the model using the mixed $R^{N}$ and ${Rrs}^{N}$ and verify the model performance using ${Rrs}^{N}$; and (**m**–**p**) train the model using the mixed $R^{N}$ and ${Rrs}^{N}$ and verify the model performance using $R^{N}$.

As shown in Figure 7, after normalizing the hyperspectral data, the performance of the SVR, RF, and 1D-CNN models under various input conditions has been improved. However, the inversion accuracy of the SVR for high-concentration COD is still low. Comparing the MAPE of all the models in Figure 7, the 1D-CNN has the best performance under all training conditions, and the MAPEs are all lower than 30%; comparing the RMSE and MAE of all the models, the RF has the best performance under all conditions, the RMSE is all lower than 5.50 mg/L, and the MAEs are all lower than 3.60 mg/L. For the SVR, RF, and BPNN, the various errors of using the mixed ${Rrs}^{N}$ and $R^{N}$ for training are all better than using the ${Rrs}^{N}$ or $R^{N}$ alone for training. For the 1D-CNN, although the MAPE of using the mixed ${Rrs}^{N}$ and $R^{N}$ for training is not as good as using the ${Rrs}^{N}$ or $R^{N}$ alone for training, there is an improvement in the RMSE and MAE.

## 4. Discussion

### 4.1. Analysis of the Comparison of Model Performance Before and After Hyperspectral Normalization

#### 4.1.1. Verifying the Model Performance Using Different Types of Hyperspectral Data

As depicted in Figure 4a–h and Figure 6a–h, regardless of whether an inversion model is constructed with R and its performance is validated with Rrs, or vice versa, the error results obtained are ultimately rather substantial. This may be due to the difference in the numerical magnitude between Rrs and R. The range of Rrs for all spectra in this study is between 0 and 0.08, while the range of R is between 0 and 0.3. This magnitude gap is removed after normalizing Rrs and R, which makes it possible for the machine learning model to accept the different types of spectral data as homogeneous input information. This enhances the model’s performance by enabling machine learning to extract and learn the most significant features in the spectra. Similarly, when R and Rrs are mixed and input into the machine learning model for training, if R and Rrs are not normalized, the magnitude gap will also affect the training process of the machine learning algorithm, thereby affecting the final model performance. Therefore, if a general inversion model is to be constructed, different hyperspectral data cannot be simply mixed directly.

#### 4.1.2. Testing the Model Performance Using the Same Type of Hyperspectral Data

**Figure 8:**
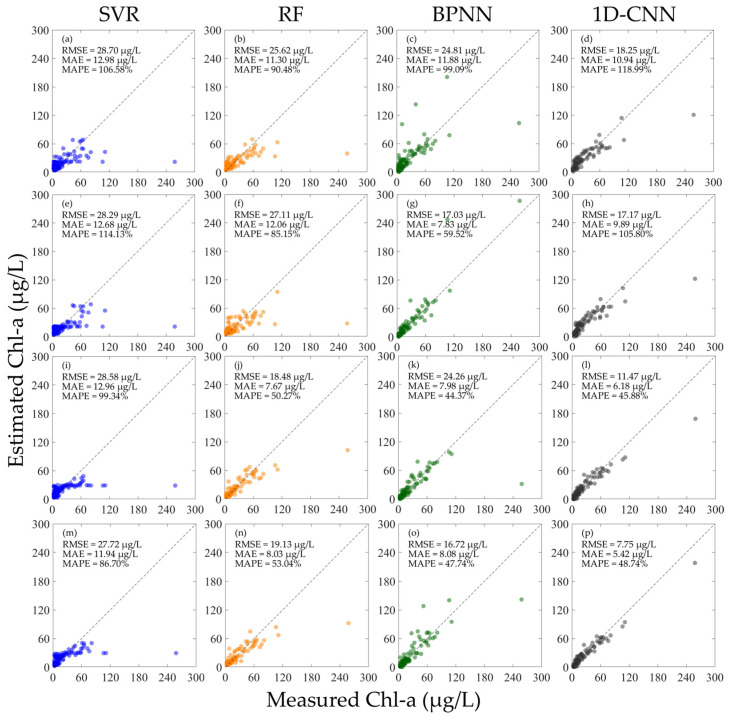
Results of testing machine learning inversion models for Chl-a concentration using the same type of hyperspectral data. (**a**–**d**) Train the model using R and test the model performance using R; (**e**–**h**) train the model using Rrs and test the model performance using Rrs; (**i**–**l**) train the model using $R^{N}$ and test the model performance using $R^{N}$; and (**m**–**p**) train the model using ${Rrs}^{N}$ and test the model performance using ${Rrs}^{N}$.

**Figure 9:**
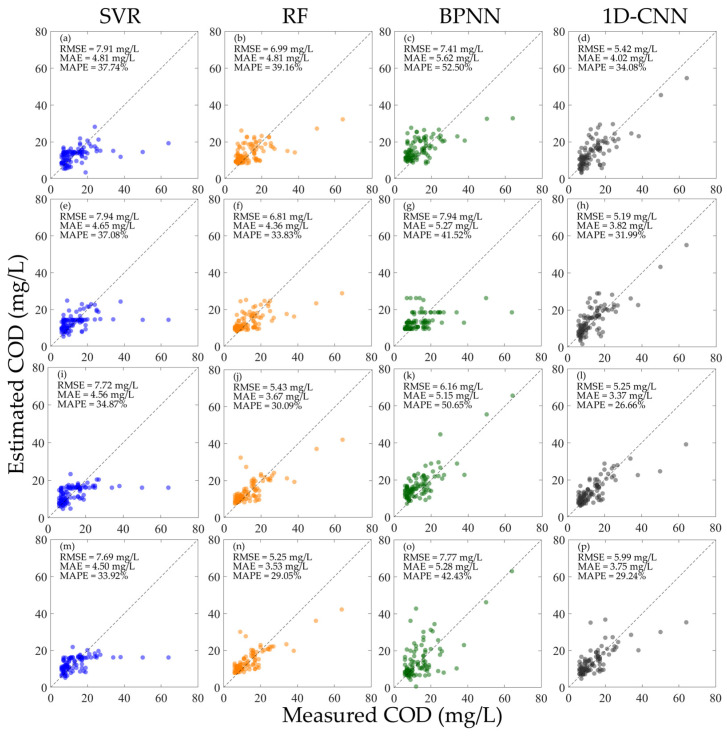
Results of testing machine learning inversion models for COD concentration using the same type of hyperspectral data. (**a**–**d**) Train the model using R and test the model performance using R; (**e**–**h**) train the model using Rrs and test the model performance using Rrs; (**i**–**l**) train the model using $R^{N}$ and test the model performance using $R^{N}$; and (**m**–**p**) train the model using ${Rrs}^{N}$ and test the model performance using ${Rrs}^{N}$.

Based on the model constructed in Section 3, this study used the same type of hyperspectral data to test the performance of the existing model, and the results are shown in Figure 8 and Figure 9.

The results show that when testing the performance of the existing model using the same type of hyperspectral data, the performance of the model constructed using Rrs outperforms the one constructed using R. The performance of the model constructed using ${Rrs}^{N}$ is generally better than that of the model constructed using $R^{N}$; and compared with directly using R or Rrs, the performance of the model constructed using $R^{N}$ or ${Rrs}^{N}$ is better, and this is particularly prominent when inverting the Chl-a concentration. Combining the results discussed in Section 4.1.1., this might offer the following insights for subsequent research on water quality monitoring in river networks based on hyperspectral data:

(1) If the original hyperspectral data remains unnormalized, it is advisable to use Rrs to construct the water quality inversion model. The results presented in Figure 4a–h, Figure 6a–h, Figure 8a–h, and Figure 9a–h suggest that, regardless of whether the model’s performance is evaluated using R or Rrs, in most scenarios, the model constructed with Rrs outperforms the one constructed with R. However, when the hyperspectral data types among different datasets are inconsistent, that is, some datasets only have R or Rrs, and some have both R and Rrs, even if R and Rrs are combined to build a model, it may still not be able to accurately invert the concentrations of Chl-a and COD.
(2) If the original hyperspectral data is normalized, whether constructing a model using either $R^{N}$ or ${Rrs}^{N}$ alone, or using both $R^{N}$ and ${Rrs}^{N}$ at the same time, a model with satisfactory performance can be obtained. Moreover, there is no necessity to deliberately differentiate the data in the training set from that in the validation set. As depicted in Figure 5, Figure 7, Figure 8i–p, and Figure 9i–p, when building models with different types of normalized hyperspectral data, the final model performance exhibits no significant disparities. This further indicates that the water quality inversion model constructed using normalized hyperspectral data demonstrates greater stability. This provides a reference for expanding the spectral dataset in the future. That is, some datasets only provide R, some datasets only provide Rrs, or some datasets provide both R and Rrs. hyperspectral normalization can fuse these different datasets to achieve the maximum utilization of data. Secondly, when it is inconvenient to measure the skylight signal, making it impossible to obtain Rrs, only the signals of the standard plate and the water body need to be measured to calculate R. Subsequently, R can be normalized.
(3) Ordinarily, multiple error metrics are employed to assess model performance, aiming to circumvent the limitations inherent in a single metric. As illustrated in Figure 5l,p,n,o, as well as Figure 8o,p, the variations in RMSE, MAE, and MAPE do not invariably remain consistent. In addition to the values of each error metric, attention should also be directed towards the scatter-plot distribution of the model, and the fitting performance of the model at extreme values should be examined. Through a comprehensive comparison of the error results of each model in this study and considering the complexity of river networks water body, perhaps the value of RMSE can be utilized as the primary error evaluation metric. However, it is also advisable not to overlook other error metrics simultaneously.

### 4.2. Analysis of the Performance Differences of Machine Learning Models

The four models used in this study exhibit significant differences in characteristics in water quality inversion, which may be closely related to their structural characteristics. As the model with the best overall performance, the advantage of 1D-CNN lies in that it can extract local spectral features through convolutional kernels and downsample the extracted features through pooling layers, effectively reducing the dimensionality of the data [42,60]. This highly coincides with the sequential attribute of hyperspectral data (wavelengths form a one-dimensional sequence), enabling it to have an advantage in capturing the subtle spectral responses of water quality parameters. RF shows the best robustness in COD inversion, with the lowest RMSE among all models. This could potentially be attributed to the way RF combines multiple decision trees through the bagging approach. This integration makes RF more stable when dealing with multi-feature data [61,62]. In contrast, SVR performs poorly in the inversion of high concentration Chl-a and COD (the MAPE of SVR is always higher than 30%). This may be because when the RBF kernel function is confronted with hyperspectral data, which may feature multiple inflection points and peaks, its non-linear fitting capabilities tend to decline [63]. Although BPNN has a strong non-linear fitting ability, when faced with high-dimensional input data, problems such as gradient disappearance and getting trapped in local optimal solutions may occur [64,65], resulting in its overall performance being inferior to RF and 1D-CNN when using normalized hyperspectral data for training.

## 5. Conclusions

After analyzing and processing the water samples collected on site, each set of Chl-a and COD concentration data corresponds to four sets of hyperspectral data, namely R, Rrs, $R^{N},$ and ${Rrs}^{N}$. This study used R, Rrs, the mixed R and Rrs, and the corresponding normalized data as inputs to establish SVR, RF, BPNN, and 1D-CNN models based on different types of hyperspectral data to invert the concentrations of Chl-a and COD, and the models’ performance was assessed using various hyperspectral data. When using the hyperspectral data before normalization for training, if different types of hyperspectral data are used to verify the model performance, the BPNN model has more advantages; if the same type of hyperspectral data is used to test the model performance, the performance of the BPNN (for Chl-a) and 1D-CNN (for COD) models is better. When using the hyperspectral data after normalization for training, regardless of whether the same type of hyperspectral data is used for testing or not, the performance of the 1D-CNN model is more stable. Overall, hyperspectral normalization processing can effectively improve the inversion accuracy of the model and can unify different hyperspectral data types.

Compared with traditional water quality monitoring methods, the hyperspectral reflectance sensor network is undoubtedly a more environmentally friendly and efficient method. When using different hyperspectral sensors or different reflectance calculation methods, normalization processing can effectively unify different data, which is more conducive to the construction and training of the model. This study provides a reference for the future construction of sensor networks for water quality monitoring.

## Figures and Tables

**Table 1:** Water quality parameter concentrations.

Parameter	Minimum	Maximum	Average	Standard Deviation
Chl-a (µg/L)	3	320	22.42	31.22
COD (mg/L)	5	72	13.30	8.76

## Data Availability

The raw data supporting the conclusions of this article will be made available by the corresponding author on request.
